# Multimodal ultrasound-based radiomics and deep learning for differential diagnosis of O-RADS 4–5 adnexal masses

**DOI:** 10.1186/s40644-025-00883-z

**Published:** 2025-05-23

**Authors:** Song Zeng, Haoran Jia, Hao Zhang, Xiaoyu Feng, Meng Dong, Lin Lin, XinLu Wang, Hua Yang

**Affiliations:** 1 Department of Ultrasound, Shengjing Hospital of China Medical University, Shenyang, China; 2 Department of Thoracic Surgery, Shengjing Hospital of China Medical University, Shenyang, China

**Keywords:** Artificial intelligence, Radiomics, Deep learning, Adnexal masses, Contrast-enhanced ultrasound, O-RADS

## Abstract

**Background:**

Accurate differentiation between benign and malignant adnexal masses is crucial for patients to avoid unnecessary surgical interventions. Ultrasound (US) is the most widely utilized diagnostic and screening tool for gynecological diseases, with contrast-enhanced US (CEUS) offering enhanced diagnostic precision by clearly delineating blood flow within lesions. According to the Ovarian and Adnexal Reporting and Data System (O-RADS), masses classified as categories 4 and 5 carry the highest risk of malignancy. However, the diagnostic accuracy of US remains heavily reliant on the expertise and subjective interpretation of radiologists. Radiomics has demonstrated significant value in tumor differential diagnosis by extracting microscopic information imperceptible to the human eye. Despite this, no studies to date have explored the application of CEUS-based radiomics for differentiating adnexal masses. This study aims to develop and validate a multimodal US-based nomogram that integrates clinical variables, radiomics, and deep learning (DL) features to effectively distinguish adnexal masses classified as O-RADS 4–5.

**Methods:**

From November 2020 to March 2024, we enrolled 340 patients who underwent two-dimensional US (2DUS) and CEUS and had masses categorized as O-RADS 4–5. These patients were randomly divided into a training cohort and a test cohort in a 7:3 ratio. Adnexal masses were manually segmented from 2DUS and CEUS images. Using machine learning (ML) and DL techniques, five models were developed and validated to differentiate adnexal masses. The diagnostic performance of these models was evaluated using the area under the receiver operating characteristic (ROC) curve (AUC), accuracy, sensitivity, specificity, precision, and F1-score. Additionally, a nomogram was constructed to visualize outcome measures.

**Results:**

The CEUS-based radiomics model outperformed the 2DUS model (AUC: 0.826 vs. 0.737). Similarly, the CEUS-based DL model surpassed the 2DUS model (AUC: 0.823 vs. 0.793). The ensemble model combining clinical variables, radiomics, and DL features achieved the highest AUC (0.929).

**Conclusions:**

Our study confirms the effectiveness of CEUS-based radiomics for distinguishing adnexal masses with high accuracy and specificity using a multimodal US-based radiomics DL nomogram. This approach holds significant promise for improving the diagnostic precision of adnexal masses classified as O-RADS 4–5.

**Supplementary Information:**

The online version contains supplementary material available at 10.1186/s40644-025-00883-z.

## Introduction

Adnexal masses are common in women, with benign masses often resolving spontaneously or managed through conservative surgical approaches [1]. In contrast, ovarian cancer (OC), the most prevalent malignant adnexal mass, typically requires aggressive treatment strategies such as cytoreductive surgery followed by chemotherapy or neoadjuvant chemotherapy prior to interval debulking [2].The prognosis of OC varies markedly between early and advanced stages, with early detection significantly improving survival rates [3]. Therefore, accurate differentiation between benign and malignant adnexal masses is essential for guiding optimal clinical management and ensuring favorable patient outcomes.

Imaging modalities such as ultrasound (US), computed tomography (CT), positron emission tomography/CT (PET/CT), and magnetic resonance imaging (MRI) are commonly used for the differential diagnosis of adnexal masses. Among these, transvaginal ultrasound (TVUS) has become the preferred diagnostic and screening tool for gynecological diseases due to its high diagnostic accuracy, cost-effectiveness, ease of use, and lack of ionizing radiation [4]. TVUS also plays a vital role in post-treatment follow-up, enabling timely detection of recurrence or complications [5]. While color Doppler US provides valuable information on blood flow, contrast-enhanced US (CEUS) offers superior visualization of tumor microvasculature and dynamic perfusion within lesions, enhancing diagnostic precision [6, 7]. Previous studies have confirmed the utility of CEUS in distinguishing between benign and malignant adnexal masses, underscoring its potential as a reliable diagnostic tool [8–11]. However, the accuracy of US-based diagnosis remains heavily dependent on the subjective interpretation and expertise of the radiologist, leading to variability in diagnostic outcomes across practitioners with differing levels of experience. Studies have demonstrated that the Ovarian-Adnexal Reporting and Data System (O-RADS) US risk stratification effectively categorizes the malignancy risk of adnexal masses, with O-RADS categories 4 and 5 representing the highest malignancy rates at 34.46% and 89.57%, respectively [12]. Given the substantial proportion of malignant cases within these categories, accurately assessing the malignancy risk of O-RADS 4 and 5 ovarian masses is critical for guiding appropriate clinical decision-making.

Radiomics refers to the high-throughput extraction and analysis of a large number of advanced and quantitative imaging features from medical imaging images. As a promising artificial intelligence (AI) technology for cancer detection, radiomics has shown a good application prospect in tumor differential diagnosis, progression prediction and treatment effect monitoring [13–16]. Machine learning (ML) and deep learning (DL) are widely utilized techniques in radiomics, and the combination of features obtained from these two methods has become increasingly popular in medical research [17, 18]. However, there is limited research on applying this approach to the diagnosis of adnexal masses, particularly using two-dimensional US (2DUS) and CEUS [19].

Given the recent advancements in the aforementioned methods, we have conducted a retrospective study with the aim of developing and validating a novel nomogram based on multimodal US, by integrating clinical variables, radiomics, and DL features, to differentiate between benign and malignant adnexal masses in O-RADS 4 and 5, thereby providing accurate diagnostic information for both patients and physicians.

## Materials and methods

### Study population and design

**Fig. 1:**
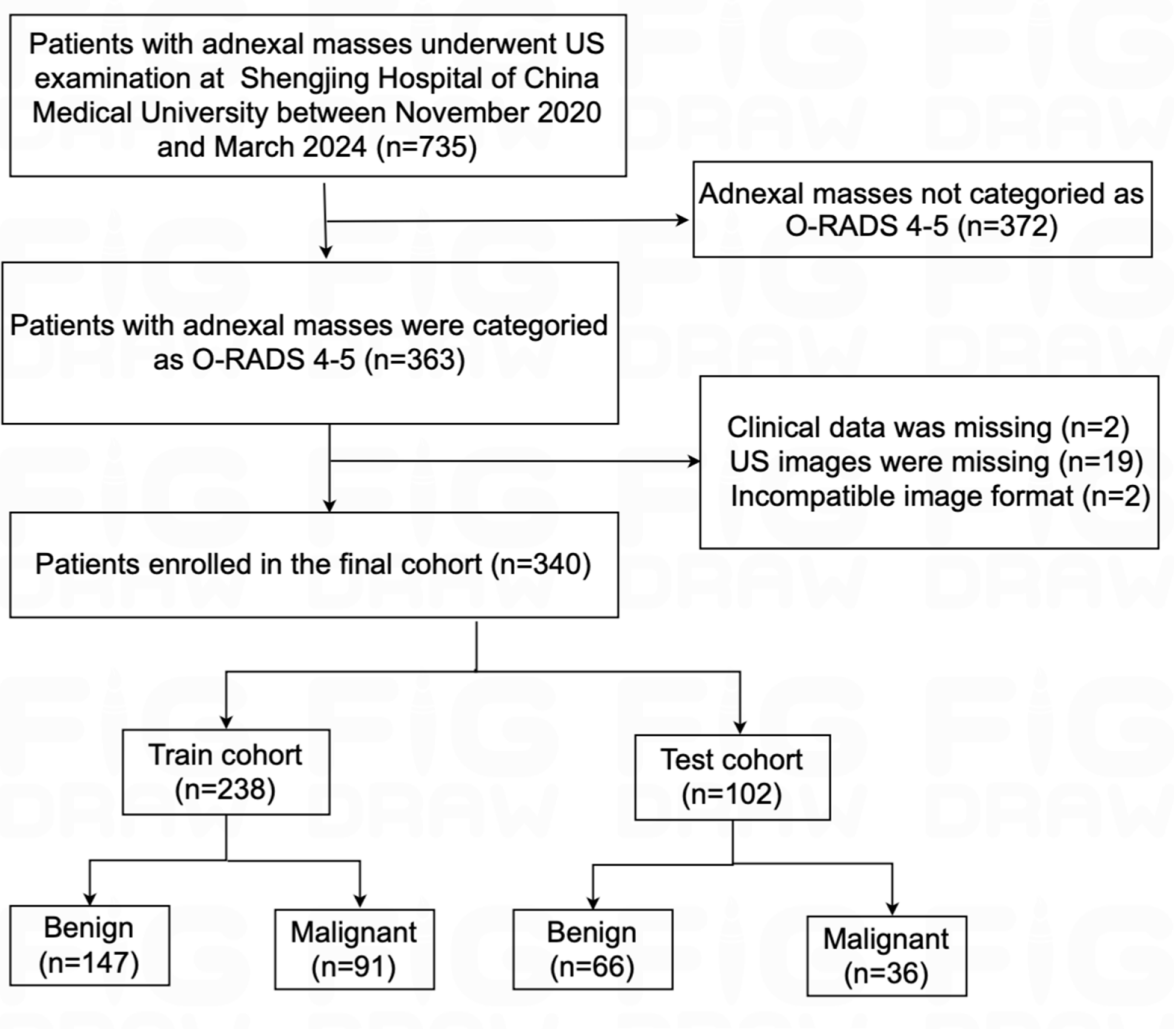
Flowchart of patient selection. US, ultrasound; O-RADS, Ovarian-Adnexal Reporting and Data System

**Fig. 2:**
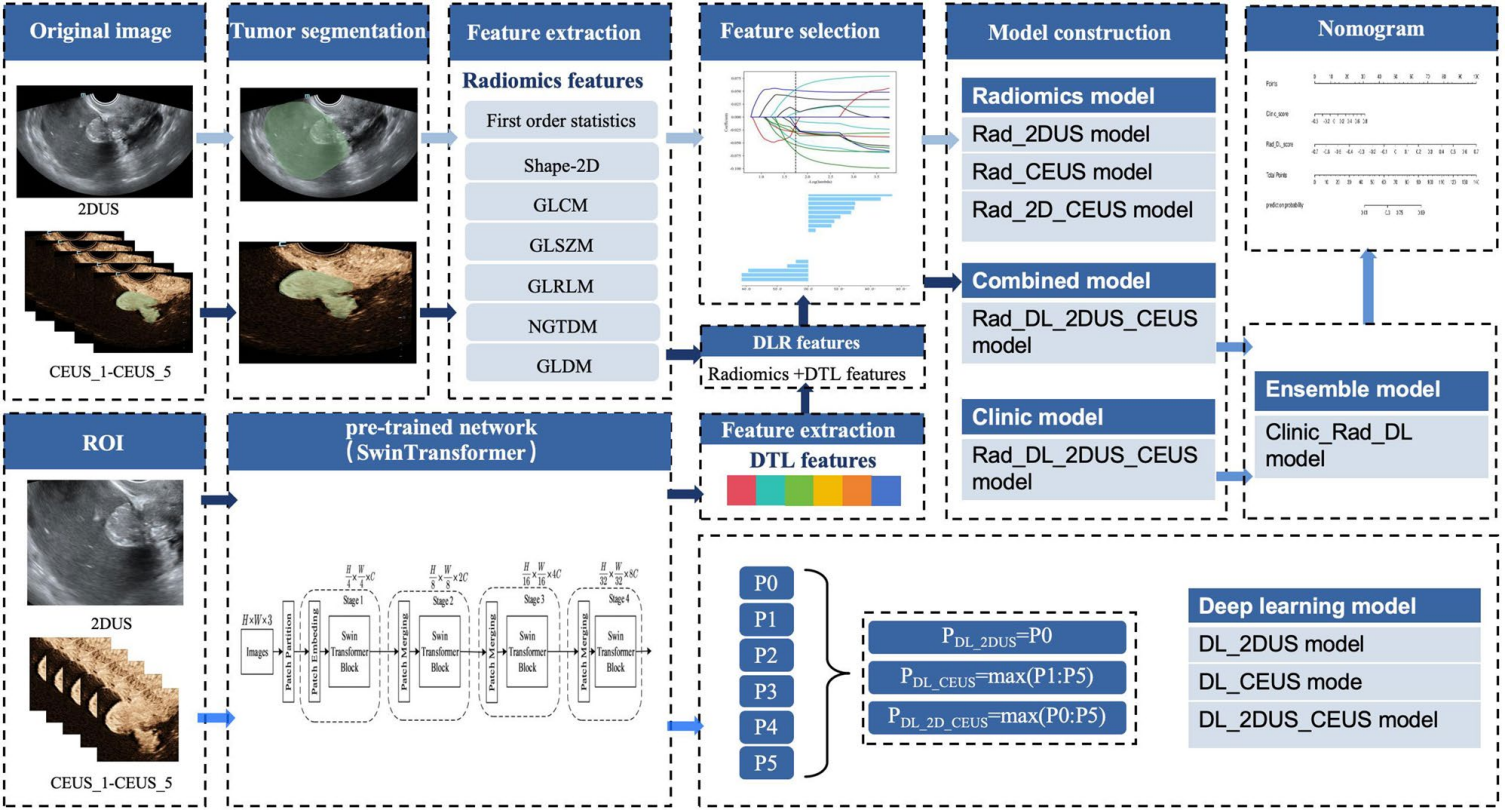
Workflow of study design. 2DUS and CEUS images were segmented, and radiomics features were extracted and selected to construct three radiomics models. Subsequently, 2DUS and CEUS images were input into a pre-trained network to construct three DL models. The radiomics features and DTL features were then selected to construct a combined model. Furthermore, an ensemble model was constructed by integrating clinical variables with radiomics and DTL features. Finally, a nomogram was developed based on the ensemble model. 2DUS, two-dimensional US; CEUS, contrast-enhanced US; DL, deep learning; DTL features, deep transfer learning features; DLR features, deep learning radiomics features

We retrospectively collected data from 735 consecutive patients with adnexal masses at Shengjing Hospital of China Medical University between November 2020 and March 2024. In the current study, inclusion criteria were as follows: (i) Patients with adnexal masses confirmed by clinical follow-up or surgical pathology; (ii) patients with adnexal masses categorized as O-RADS 4–5 by US. Exclusion criteria were as follows: (i) absence of clinical or pathological data; (ii) absence of US images; (iii) poor image quality of US, or images not in digital imaging and communications in medicine (DICOM) format. Among the cases collected, borderline adnexal masses were classified as malignant [20]. In addition, the adnexal masses that have not undergone surgical intervention are classified as benign if they have received conservative treatment for a duration of six months or longer, without any evidence of progression observed in subsequent US follow-up assessments [21]. Ultimately, 340 patients were selected and included in this study, and the patient flowchart is presented in Fig. 1. All participants were randomly divided into a train cohort and a test cohort in a ratio of 7:3. The workflow of this study is illustrated in Fig. 2.

### Clinical data

The clinical data for all patients were obtained from electronic medical records including the age of patients and serum indicators such as the carcinoembryonic antigen (CEA)、carbohydrate antigen 125 (CA125)、CA199、CA724, human epididymis protein 4 (HE4).

### Ultrasound examination

All US examinations were performed by experienced sonographers specializing in gynecological oncological US and CEUS. The enrolled participants underwent US using a Mindray Resona R9 (Mindray Co., Ltd, Shenzhen, China) US machine. A DE10-3WU probe with a frequency range of 3–10 MHz and a SC6-1U probe with a frequency range of 1–6 MHz were used. All participants underwent transvaginal US whenever feasible. Transabdominal US was used in cases where the tumor size prevented complete visualization using transvaginal US. Transrectal or transabdominal US was used if the patient was unsuitable for a transvaginal US examination. Only one adnexal mass was chosen per patient. In cases where more than one mass was detected, the mass with the highest category or, if categories were similar, the largest one was utilized. The size, shape, edge, internal echogenicity, ascites, vascularity, and arterial spectral resistance index (RI) were recorded. The largest solid part of the lesion was selected as the section of interest for contrast-enhanced observation. A bolus of 1.6–2.0 mL of the sulfur-hexafluoride contrast agent (SonoVue; Bracco, Milan, Italy) was rapidly injected into the antecubital vein, followed by a 5-mL saline flush. The real-time enhancement mode of the contrast agent in the lesion was observed for approximately 1.5 min after the injection, and the video was recorded in DICOM format. Average contrast signal intensity (MeanLin), peak enhancement (PE), time to peak (TTP), and wash-in rate (WiR) derived from time-signal intensity curve (TIC) were recorded. These parameters were categorized as 1 or 2 based on the absolute value of the lesion itself or the relative value of the lesion and normal myometrium; for example, MeanLin1 represents the average contrast signal intensity of the lesion itself, while MeanLin2 denotes the average contrast signal intensity of the lesion in relation to the normal myometrium.

Two sonographers (XL. W., H. Y.; 20 and 17 years of experience in gynecologic US, respectively) interpreted all US images and assigned them to an O-RADS category for each adnexal mass according to the O-RADS US risk stratification and management published by American College of Radiology (ACR) [22]. When confronted with ambiguous adnexal mass, the mass was reevaluated until a consensus was reached.

Prior to the analysis, the US images underwent a selection process. One 2DUS image of the largest section of the mass from DICOM video was captured. Additionally, a total of five CEUS images were captured from the DICOM video. One image was taken at the time of the peak of the contrast agent according to the TIC. Subsequently, one image was taken every 2 s before and after the peak, resulting in two additional images each.

### Tumor segmentation

For each adnexal mass, the region of interest (ROI) was manually delineated by radiologist S. Z. using 3D slicer software (www.slicer.org) and then validated by radiologist H. Y. In cases of disagreements, the decision was made by the more senior radiologist XL. W. The entire masses were delineated on the 2DUS images, while the solid part of them, which was filled with the contrast agent, was delineated on the CEUS images. To assess the reproducibility of extracted radiomics features and to obtain more robust radiomics features, the intra-class correlation coefficient (ICC) was used to evaluate both intra- and inter-observer agreement in ROI delineation.

### Extraction and selection of radiomics features

Pyradiomics (version 3.0.1, https://pyradiomics.readthedocs.io/en/latest/) was employed for feature extraction based on segmented lesion regions, resulting in the extraction of 846 radiomics features for each image. The types of features include first-order features, 2D shape features, gray level cooccurrence matrix (GLCM), gray level size zone matrix (GLSZM), gray level run length matrix (GLRLM), neighborhood gray tone difference matrix (NGTDM), and gray level dependence matrix (GLDM). Radiomics features were extracted from a single 2DUS image to generate a 2DUS feature set and were also extracted from five CEUS images and fused to obtain a CEUS feature set. All the aforementioned radiomics features were combined to generate a 2DUS-CEUS feature set. Z-Score normalization was utilized for standardizing the train cohort and test cohort with respect to the extracted radiomics features. Additionally, a pre-trained DL network, Swin Transformer, trained on the ImageNet dataset (http://www.image-net.org), was applied as a feature extractor. This resulted in obtaining 768 DL features from each image, which were then fused with the features obtained from a single 2DUS image and five CEUS images to obtain a DL feature set.

The features of the patients in the train cohort were selected by four steps. Firstly, features with an ICC > 0.8 were chosen for further analysis. Secondly, the variance threshold method was applied to select features with a variance greater than 0.75. Next, the univariate selection method SelectKBest was used to assess the relationship between features and classification results, selecting those with *p* < 0.05. Finally, the least absolute shrinkage and selection operator (LASSO) algorithm with 10-fold cross-validation was utilized to determine the feature and its coefficient by using mean square error (MSE) to establish the parameter λ and retain features whose coefficient is not zero.

### Model construction

LASSO with 10-fold cross-validation was utilized to determine the clinical variables (including serum indicators and US reported data) and their coefficients. The clinical model (Clinic_model) was constructed based on clinical scores, which were calculated by linear weighting of the final selected clinical features according to their respective characteristic regression coefficients. The formula is as follows: ${\rm{score}} = Intercept + \sum\limits_{i = 1}^n {{\rm{coefficients}}[i] \times } Feature[i]$

By utilizing radiomics scores, which were determined through linearly weighting the final features selected from various radiomics feature sets based on their respective characteristic regression coefficients, we were able to develop radiomics models. These included the radiomics 2DUS model (Rad_2DUS model), Rad_CEUS model, and Rad_2D_CEUS model using four classifiers: K-nearest neighbor (KNN), support vector machine (SVM), logistic regression (LR), and random forest (RF).

Based on the theory of transfer learning (TL), we selected the Swin Transformer DL network, which was pre-trained on the ImageNet dataset, as the foundational model for the DL model in this study. The Swin Transformer utilized the Patch Partition image segmentation module to divide the RGB image of H×W into independent patches, with each patch’s feature being a series of the original pixel RGB values. The Patch Embeding maps these original feature values to any dimension and ultimately processes the image size as ${H \over 4} \times {W \over 4} \times C$. After each Patch Merging layer, both H and W dimensions of the image were reduced to half of their previous sizes, while the feature channel was expanded by 2 times (Supplementary data, Figure S1). We initially cropped out ROIs from 2DUS and CEUS images and normalize pixels to a range between 0 and 255. All input images were converted to 3-channel RBG format and resized to a size of 224 × 224 × 3. Subsequently, we input the ROIs of 2DUS images, the ROIs of CEUS images, and the ROIs combining both into the network; then we used the output probability from the network as our classification result. As such, different DL models were obtained, including DL_2DUS model, DL_CEUS model and DL_2D_CEUS model.

We have developed a combined model (Rad_DL_2D_CEUS model) based on radiomics scores derived from both 2D-CEUS and DL feature sets; additionally, an ensemble model (Clinic_Rad_DL model) has been created using clinical variables along with both 2D-CEUS feature set and DL feature set employing four classification methods. Finally, a nomogram model fusing the clinical, radiomics, and DL features was established for final interpretation and analysis. The nomogram was constructed using multivariate logistic regression to combine the scores of these features developed on the training cohort. A nomogram score was then calculated for each patient in both the training and test cohorts to predict the risk of malignancy with this score combining the Clinic_score and Rad_ DL_ score weighted by their respective coefficients.

### Model evaluation

The model performance was assessed using the area under the receiver operating characteristic (ROC) curve (AUC), accuracy, sensitivity, specificity, precision, and F1-score. The DeLong test was employed to compare differences in diagnostic performance between models. A calibration curve was generated to assess model fit. Decision curve analysis (DCA) was utilized to quantitatively evaluate the overall benefit of the predictive model across various threshold probabilities.

### Statistical methods

The normality of continuous data was assessed using the Kolmogorov-Smirnov test. For measurement data that followed a normal distribution, the t-test was employed, while the Mann-Whitney U test was used for non-normally distributed data. Categorical variables were analyzed using either the chi-square test or Fisher’s exact test. A two-tailed significance level of *p* < 0.05 was considered statistically significant. All statistical analyses were conducted using R software (version 4.2.3 https://www.r-project.org) and SPSS (version 25.0; IBM).

## Results

### The clinical baseline data

A total of 340 eligible patients were enrolled in the current study. The train cohort comprised 238 patients, with 147 being benign and 91 malignant. The test cohort included 102 patients, with 66 being benign and 36 malignant. Table 1 presents a comparison of the baseline clinical characteristics of the patients between the two cohorts including the age of patients, serum indicators such as CEA, CA125, CA199, CA724, HE4, size and edge of the mass, presence or absence of solid component and ascites, and quantitative indicators of CEUS including MeanLin1/2, PE1/2, TTP1/2, and WiR1/2.

**Table 1:** Baseline clinical characteristics of participants between two cohorts

	Train			Test			*P*-value
Variables	Benign (*n* = 147)	Malignant (*n* = 91)	P-value	Benign (*n* = 66)	Malignant (*n* = 36)	P-value	
Age, Median (Q1, Q3)	45 (34, 57)	52 (39, 60.5)	0.023	43.1 ± 13	54.7 ± 13.5	< 0.001	0.875
CEA, n (%), µg/L			0.002			0.051	0.766
< 5	146 (99.3)	83 (91.2)		65 (98.5)	32 (88.9)		
≥ 5	1 (0.7)	8 (8.8)		1 (1.5)	4 (11.1)		
CA125,n (%), U/mL			< 0.001			< 0.001	0.897
< 35	127 (86.4)	40 (44)		60 (90.9)	13 (36.1)		
≥ 35	20 (13.6)	51 (56)		6 (9.1)	23 (63.9)		
CA199, n (%), U/mL			0.009			0.049	1.000
< 37	137 (93.2)	74 (81.3)		62 (93.9)	29 (80.6)		
≥ 37	10 (6.8)	17 (18.7)		4 (6.1)	7 (19.4)		
CA724, n (%), U/mL			< 0.001			0.077	0.889
< 6.9	125 (85)	58 (63.7)		54 (81.8)	23 (63.9)		
≥ 6.9	22 (15)	33 (36.3)		12 (18.2)	13 (36.1)		
HE4, n (%), pmol/L			< 0.001			< 0.001	0.908
< 140	145 (98.6)	67 (73.6)		66 (100)	26 (72.2)		
≥ 140	2 (1.4)	24 (26.4)		0 (0)	10 (27.8)		
Size, n (%), cm			0.006			0.001	0.741
< 5	71 (48.3)	27 (29.7)		35 (53)	11 (30.6)		
5–10	56 (38.1)	40 (44)		27 (40.9)	13 (36.1)		
> 10	20 (13.6)	24 (26.4)		4 (6.1)	12 (33.3)		
Edge, n (%)			< 0.001			0.001	0.914
Clear	130 (88.4)	48 (52.7)		56 (84.8)	19 (52.8)		
Not clear	17 (11.6)	43 (47.3)		10 (15.2)	17 (47.2)		
Solid component, n (%)			0.120			0.539	0.099
No	14 (9.5)	3 (3.3)		2 (3)	0 (0)		
Yes	133 (90.5)	88 (96.7)		64 (97)	36 (100)		
Ascites, n (%)			< 0.001			< 0.001	0.934
No	143 (97.3)	74 (81.3)		65 (98.5)	27 (75)		
Yes	4 (2.7)	17 (18.7)		1 (1.5)	9 (25)		
MeanLin1, Median (Q1, Q3)	19266.9 (7764.8, 60711.5)	45210.4 (19849.9, 131700.7)	< 0.001	23143.9 (10248.1, 64098.2)	75201.9 (31500.9, 211904.9)	< 0.001	0.327
MeanLin2, Median (Q1, Q3)	21.9 (10, 64.1)	50.8 (32.7, 95.3)	< 0.001	19.8 (8.3, 59.1)	66.8 (29.4, 199)	< 0.001	0.832
PE1, Median (Q1, Q3)	40654.6 (12796.2, 128684.4)	134167.2 (53520.6, 406947.7)	< 0.001	51599.1 (14543.9, 145187.4)	203084.8 (69860.8, 515958.1)	< 0.001	0.511
PE2, Median (Q1, Q3)	18.5 (7.8, 57.8)	51 (31.2, 102.4)	< 0.001	20.8 (5.9, 52.2)	77.2 (32.6, 176.7)	< 0.001	0.831
TTP1, Median (Q1, Q3)	16 (11.8, 21.1)	13.3 (11.4, 16)	0.003	15.1 (11.7, 21.6)	15.3 (12.7, 21.2)	0.729	0.19
TTP2, Median (Q1, Q3)	126 (84.6, 173)	101.3 (86, 132.3)	0.024	114.6 (89, 186.3)	98.7 (78, 119.4)	0.069	0.858
WiR1, Median (Q1, Q3)	5671.1 (1857.7, 18789.1)	23077.8 (7559.6, 61849.3)	< 0.001	5569.6 (1580.3, 22786.6)	30801.4 (7781.2, 71827)	< 0.001	0.896
WiR2, Median (Q1, Q3)	18 (5.7, 53.3)	51.7 (21.5, 134.7)	< 0.001	15.7 (3.6, 54.7)	89.2 (27.7, 158.6)	< 0.001	0.875

Edge, solid component, ascites 0 mean the masses were ill-defined, had no solid component, and the patients had no ascites. CEA (carcinoembryonic antigen), CA125 (carbohydrate antigen 125), human epididymis protein 4 (HE4), MeanLin (Average contrast signal intensity), PE (peak enhancement), TTP (time to peak), WiR (wash-in rate)

### The selection of clinical features and construction of clinical model

**Fig. 3:**
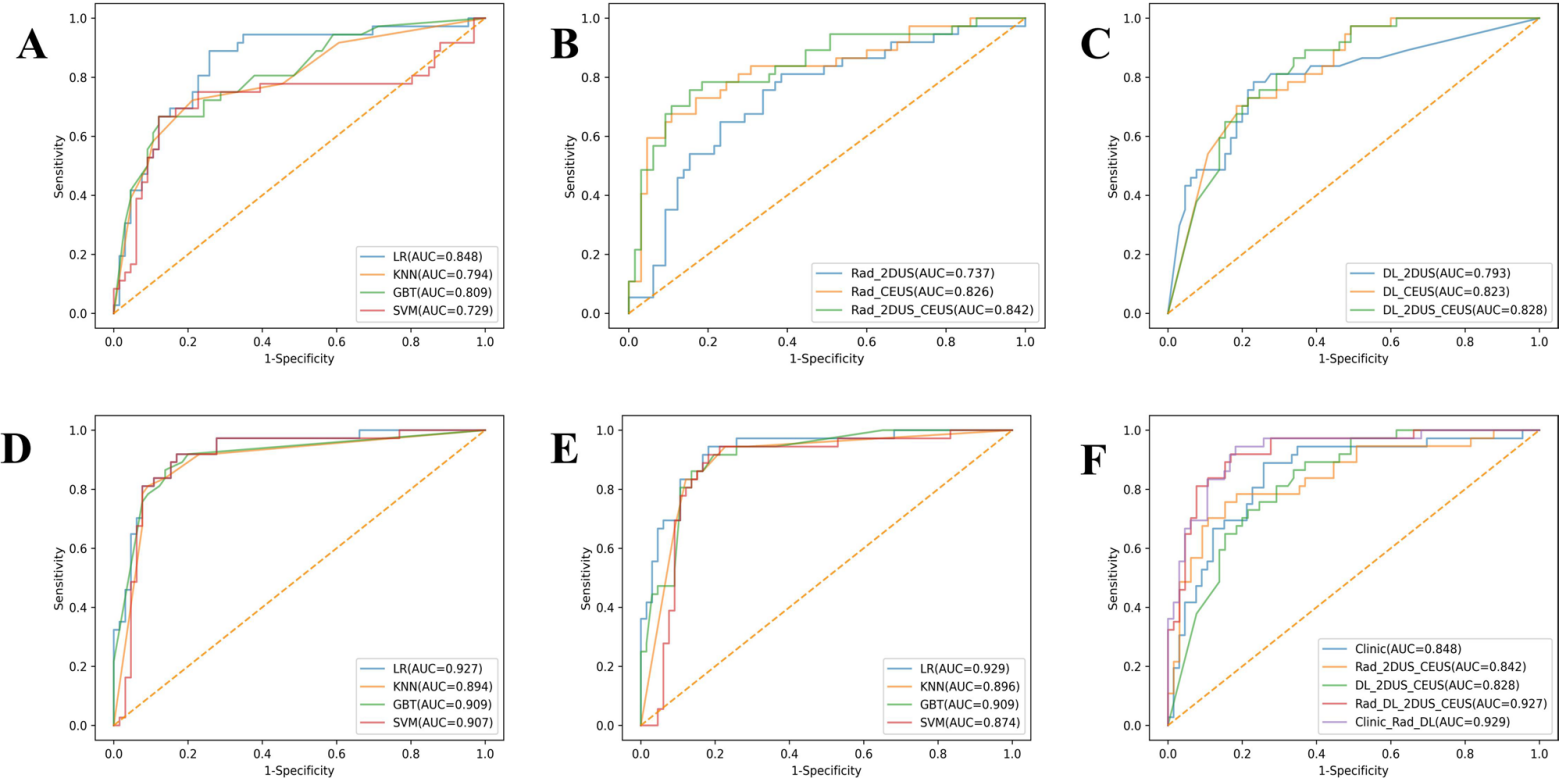
The ROC curves of models in the test cohort. (**A**) The ROC curves of Clinic_models by four classifiers. (**B**) The ROC curves of Rad_2DUS model, Rad_CEUS model, and Rad_2D_CEUS model. (**C**) The ROC curves of DL_2DUS model, DL_CEUS model, and DL_2D_CEUS model. (**D**) The ROC curves of Rad_DL_2D_CEUS models by four classifiers. (**E**) The ROC curves of Clinic_Rad_DL models by four classifiers. (**F**) The ROC curves of Clinic_model, Rad_2D_CEUS model, DL_2D_CEUS model, Rad_DL_2D_CEUS model, and Clinic_Rad_DL model. CEUS, contrast-enhanced ultrasound; 2DUS, two-dimensional ultrasound; Rad, radiomics; DL, deep learning; KNN, K-nearest neighbor; SVM, support vector machine; LR, logistic regression; RF, random forest; AUC, area under the receiver operating characteristic curve. ROC, area under the receiver operating characteristic curve

During the feature selection of clinical baseline data, the LASSO method retained 11 clinical features that effectively distinguish between benign and malignant adnexal masses (Supplementary data, Figure S2). Subsequently, Clinic_models were constructed using four classifiers - KNN, SVM, LR, and RF. Among these, LR demonstrated the best performance with an AUC of 0.833 (95% CI: 0.775–0.891) in the train cohort and 0.848 (95% CI: 0.767–0.930) in the test cohort (Fig. 3A and Supplementary data, Table S1).

### The extraction and selection of radiomics features and construction of radiomics model

The feature sets of 2DUS, CEUS, and 2DUS-CEUS were created using 846, 4230, and 5076 features extracted by pyradiomics, respectively. After a series of feature selections, 21, 17, and 31 features were preserved (Supplementary data, Table S2 and Figure S3). Subsequently, the Rad_2DUS model, Rad_CEUS model, and Rad_2D_CEUS model were developed using four classifiers. Among them, the Rad_2D_CEUS model based on LR showed the best performance with the AUCs of 0.893 (95% CI: 0.852–0.933) and 0.842 (95% CI: 0.758–0.926) in the train and test cohorts, respectively (Fig. 3B and Supplementary data, Table S3).

### Construction of deep learning model

The 2DUS, CEUS, and aforementioned images of the patients were individually input into the network to obtain three DL models: DL_2DUS model, DL_CEUS model, and DL_2D_CEUS model. The detailed performance of these DL models on both the train and test cohorts is presented in Supplementary data, Table S4. The best performing model was found to be the DL_2D_CEUS model, which achieved AUCs of 1.000 (95% CI: 1.000–1.000) and 0.828 (95% CI: 0.750–0.906) in the train and test cohorts, respectively (Fig. 3C).

### The extraction and selection of deep learning features and construction of combined model

A DL feature set comprising 4608 features was generated through feature extraction using the Swin Transformer network. This set was combined with the 2DUS-CEUS feature set, resulting in the preservation of 77 features after a series of feature selection steps (Supplementary data, Table S5 and Figure S4). Four types of classifiers were employed, among which the LR-based Rad_DL_2D_CEUS model demonstrated superior performance with the AUCs of 0.993 (95% CI: 0.984-1.000) and 0.927 (95% CI: 0.875–0.979) in the train and test cohorts, respectively (Fig. 3D and Supplementary data, Table S6).

### Construction of ensemble model

**Fig. 4:**
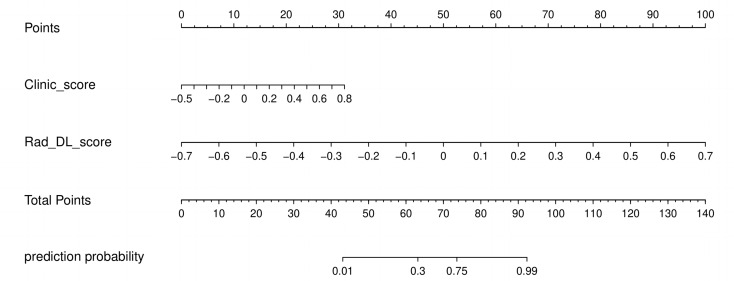
The nomogram integrating the prediction results of Clinic_score and Rad_DL_score. Rad, radiomics; DL, deep learning

An ensemble model refers to a model that incorporates clinical variables, radiomics, and DL features that have been carefully selected. Four types of classifiers were utilized, among which the LR-based Clinic_Rad_DL model demonstrated the best performance with the AUCs of 0.991 (95% CI: 0.982-1.000) and 0.929 (95% CI: 0.877–0.980) in the train and test cohorts, respectively (Fig. 3E and Supplementary data, Table S7). Furthermore, the nomogram based on the ensemble model was developed for the visualized outcome measure by combining Clinic_score and Rad_DL_score, which were weighted by their respective coefficients, to get the total score on the malignant ratio of patients (Fig. 4 and Supplementary data, Table S8).

### Model evaluation

**Fig. 5:**
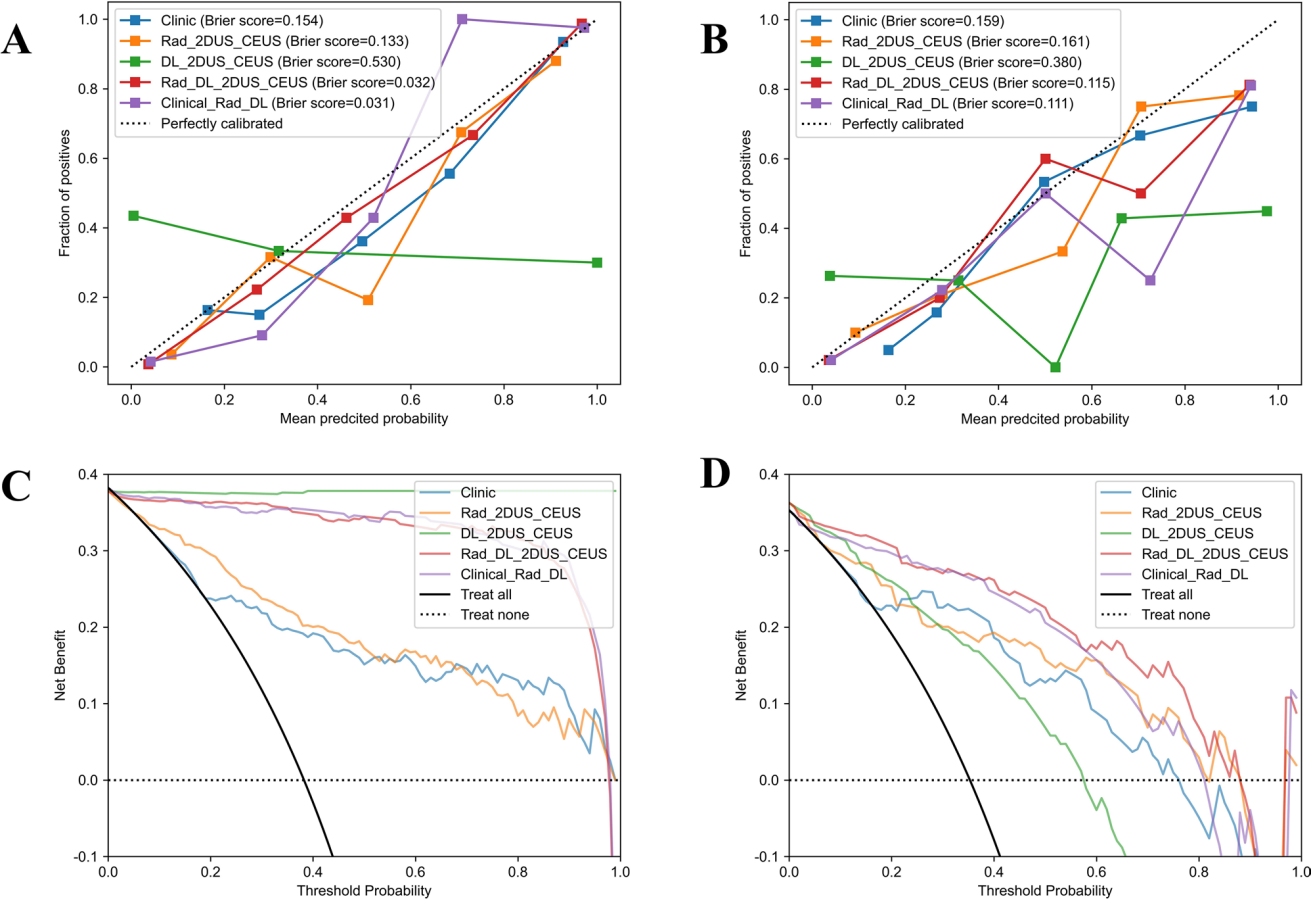
(**A**, **B**) Calibration curves of the five models in the train and test cohorts. (**C**, **D**) Decision curve analysis of the five models in the train and test cohorts. CEUS, contrast-enhanced ultrasound; 2DUS, two-dimensional ultrasound; Rad, radiomics; DL, deep learning

The diagnostic performance of five models, including the Clinic_model, Rad_2D_CEUS model, DL_2D_CEUS model, Rad_DL_2D_CEUS model, and Clinic_Rad_DL model, was presented in Fig. 3F and Supplementary data, Table S9. It was found that the Clinic_Rad_DL model exhibited the best performance in the test set with AUC of 0.929, accuracy of 0.853, sensitivity of 0.899, specificity of 0.833, precision of 0.744, and F1 score of 0.810. The Delong test revealed that the Clinic_Rad_DL model had superior diagnostic performance compared to Clinic, Rad_2DUS_CEUS, and DL_2DUS_CEUS models (*p* < 0.05), but there was no statistical difference between Clinic_Rad_DL and Rad_DL_2DUS_CEUS models (*p* = 0.394) in test cohorts (Supplementary data, Table S10). The Brier score is an indicator of calibration curve performance, which measures the difference between predicted and true values. The Clinic_Rad_DL model demonstrated good performance with a Brier score of 0.111 (Fig. 5). The DCA results are shown in Fig. 5, indicating that the Clinic_Rad_DL model provided clinical benefit within the threshold range of 0-0.97 in the train cohort and 0-0.83 in the test cohort, with maximum net benefits of 0.38 and 0.36, respectively.

## Discussion

Early differential diagnosis of adnexal masses is crucial for optimizing patient treatment outcomes [23, 24]. Research has demonstrated that utilizing O-RADS 4 as a threshold for diagnosing malignant adnexal masses resulted in a sensitivity of 99% and a specificity of 70% [25]. Given the high malignancy risk associated with O-RADS 4 and 5 masses, our study specifically focused on distinguishing between benign and malignant cases within these categories, unlike previous research that encompassed all adnexal masses. The nomogram we developed demonstrates robust diagnostic performance, achieving an AUC of 0.929 in the test cohort. This tool offers significant value in supporting clinical decision-making, enabling physicians to make more informed and precise management choices for patients with high-risk adnexal masses.

Multiple studies have been published regarding the use of radiomics models for diagnosing adnexal masses. Zhang et al. utilized a ML approach to extract 1714 features from four MRI protocols for each lesion and constructed a radiomics model to distinguish between benign and malignant adnexal masses [26]. Similarly, Li et al. employed ML to create two models a radiomics model and a mixed model incorporating three clinical predictors (HE 4, ascites, and margin) which demonstrated reliable differentiation of benign and malignant adnexal masses [27]. While DL is increasingly prevalent in radiomics, it requires large datasets for training. TL, a type of pre-trained convolutional neural network (CNN), can efficiently address this issue and mitigate overfitting caused by limited training data [28]. Additionally, TL enhances the performance of models trained on small samples by leveraging prior knowledge obtained from similar classification tasks [29]. Christiansen F. et al. ‘s DL model, based on grayscale and power doppler US, can accurately predict ovarian malignancy and is comparable to human expert examiners [30]. Similarly, Chen et al. demonstrated that the diagnostic efficacy of a DL model based on gray scale and color doppler US for ovarian tumors was comparable to expert subjective assessment and O-RADS assessment [31]. Gao et al. constructed a DL model of US based on multi-center and large-sample data, showing that its diagnostic performance exceeded the average diagnostic level of radiologists while verifying that it could enhance the diagnostic accuracy of radiologists [32]. All these studies demonstrated that TL-based DL can play an important role in identifying ovarian tumors. In the current study, DL models based on 2DUS feature set, CEUS feature set, and 2DUS-CEUS feature set were constructed, respectively. All of them showed excellent discrimination ability, among which the DL_2D_CEUS model had the best performance with an AUC of 1.000 in the train cohort and 0.828 in the test cohort. These results implied that the TL-based DL model had strong generalization capabilities and outstanding prediction performance in the differential diagnosis of adnexal masses.

CEUS is a straightforward, non-invasive imaging technique that offers exceptional spatial and temporal resolution for evaluating microcirculatory perfusion in adnexal tumors. It has been proven to improve the diagnostic performance for malignancy of the O-RADS categories 3–5 [33]. While CEUS-based radiomics has demonstrated superior performance in diagnosing various tumors [34, 35], its application to adnexal masses remains underexplored. Unlike previous studies that focused solely on a single CEUS image at the peak of contrast agent arrival [34], our study expanded the analysis to include two additional images before and after the peak time, resulting in a total of five CEUS images analyzed. This approach allowed for a more comprehensive assessment of tumor characteristics. Our findings demonstrated that the Rad_CEUS model outperformed the Rad_2DUS model, achieving AUCs of 0.826 and 0.737 in the test dataset, respectively. Furthermore, the Rad_2DUS_CEUS model exhibited superior performance with an AUC of 0.842. The integration of CEUS images enhanced the performance of conventional radiomics models based on 2DUS by providing clearer visualization of tumor microcirculation and stronger indicators of malignancy in adnexal masses. These conclusions were further corroborated by our DL model analysis, underscoring the value of CEUS in improving diagnostic precision for adnexal masses.

This study represents the first application of ML combined with radiomics and DL features extracted from 2DUS and CEUS images to differentiate between benign and malignant adnexal masses. Similar to our study, previous studies have demonstrated that an integrated model incorporating both radiomics features and DL features can significantly improve prediction accuracy and reliability, outperforming the use of either radiomics features or DL features alone [17, 36, 37]. However, research on the application of this method in adnexal masses is limited, particularly based on 2DUS and CEUS. Our combined model achieved an AUC of 0.927, surpassing the separate radiomics and DL models, which had AUCs of 0.842 and 0.828 in the test cohort, respectively. To identify the optimal model, we developed and compared five models: Clinic_model, Rad_2DUS_CEUS model, DL_2DUS_CEUS model, combined model, and ensemble model combining clinical variables with radiomics and DL features. It is noteworthy that we integrated quantitative indicators of CEUS into clinical factors to enhance diagnostic ability due to their high objectivity. The results indicated that the ensemble model exhibited superior diagnostic performance compared to other models for classifying adnexal masses, suggesting that the combination of these three types of features is particularly advantageous for distinguishing between benign and malignant lesions. Furthermore, we constructed a nomogram to visually represent the optimal model as a scale for convenient clinical application.

Despite the significant findings mentioned above, the present study has certain limitations. Firstly, this study was a single-center retrospective study with a relatively small sample size, which may limit the generalizability of the results. Another limitation is that only Chinese patients were included in this study. Variations in genetic backgrounds, lifestyles, and environmental exposures among different ethnic groups may influence the manifestation and characteristics of adnexal masses. This ethnic homogeneity of our sample might introduce bias and restrict the generalizability of our findings to other populations. Future research should aim to include patients from diverse ethnic backgrounds to enhance the applicability of the results across various demographics. To enhance and assess the model’s performance, larger datasets from multiple institutions should be utilized in future prospective study designs. Secondly, borderline masses were classified as malignant masses in this study without further classification. Thirdly, the diagnostic performance of the model was not compared with that of radiologists, and whether the model could improve the diagnostic ability of radiologists was not explored. Fourthly, manual segmentation of lesion boundaries may lead to human error and thus potentially miss useful features of the image. Although an ICC test was performed, it is necessary to establish an automatic image segmentation tool. Additionally, while we segmented the entire mass on 2DUS images and only segmented the solid part on CEUS images due to contrast agent filling only in the solid part containing microscopic information, it is still inconclusive which segmentation method is most beneficial for constructing the model and further comparison is needed.

## Conclusion

In summary, the multimodal US-based ensemble model, combining radiomics and DL features with clinical variables, provides reliable assessment of adnexal masses, and may have the potential for identifying benign and malignant adnexal masses. Integrating this tool into clinical decision-making processes is anticipated to make significant advancements in precise diagnosis of adnexal masses and individualized treatments.

## Electronic supplementary material

Below is the link to the electronic supplementary material.

Supplementary Material 1: Figure S1 The architecture of the Swin Transformer DL network. W-MSA, window multi self-attention; SW-MSA, shifted window multi self-attention; MLP, multilayer perceptron; LayerNorm, layer normalization; ML, machine learning

Supplementary Material 2: Figure S2 Clinical feature selection using the LASSO logistic regression method. MSE, mean square error; LASSO, least absolute shrinkage and selection operator

Supplementary Material 3: Figure S3 Radiomics feature selection using the LASSO logistic regression method of Rad_2DUS model, Rad_CEUS model, and Rad_2D_CEUS model. CEUS, contrast-enhanced ultrasound; 2DUS, two-dimensional ultrasound; Rad, radiomics; MSE, mean square error; LASSO, least absolute shrinkage and selection operator

Supplementary Material 4: Figure S4 Radiomics and DL features selection using the LASSO logistic regression model. MSE, mean square error, LASSO, least absolute shrinkage and selection operator

Supplementary Material 5: Table S1 Diagnostic performance of Clinic_models by four classifiers

Supplementary Material 6: Table S2 Features selection process of Rad_2DUS model, Rad_CEUS model, and Rad_2D_CEUS model

Supplementary Material 7: Table S3 Diagnostic performance of Rad_2DUS model, Rad_CEUS model, and Rad_2D_CEUS model by four classifiers

Supplementary Material 8: Table S4 Diagnostic performance of DL_2DUS model, DL_CEUS model, and DL_2D_CEUS model

Supplementary Material 9: Table S5 Features selection process of Rad_DL_2DUS_CEUS model

Supplementary Material 10: Table S6 Diagnostic performance of Rad_DL_2D_CEUS models by four classifiers

Supplementary Material 11: Table S7 Diagnostic performance of Clinic_Rad_DL models by four classifiers

Supplementary Material 12: Table S8 Features and coefficients of Clinic_model and Clinic_Rad_DL model

Supplementary Material 13: Table S9 Diagnostic performance of Clinic_model, Rad_2D_CEUS model, DL_2D_CEUS model, Rad_DL_2D_CEUS model, and Clinic_Rad_DL model

Supplementary Material 14: Table S10 The DeLong test of models

## Data Availability

No datasets were generated or analysed during the current study.

## Abbreviations

2DUS: Two-dimensional US
ACR: American college of radiology
AI: Artificial intelligence
AUC: Area under the receiver operating characteristic curve
CA: Carbohydrate antigen
CEUS: Contrast-enhanced ultrasound
CT: Computed tomography
DICOM: Digital imaging and communications in medicine
DL: Deep learning
GLCM: Gray level cooccurrence matrix
GLDM: Gray level dependence matrix
GLRLM: Gray level run length matrix
GLSZM: Gray level size zone matrix
ICC: Intra-class correlation coefficient
KNN: K-nearest neighbor
LASSO: Least absolute shrinkage and selection operator
LR: Logistic regression
MeanLin: Average contrast signal intensity
ML: Machine learning
MRI: Magnetic resonance imaging
MSE: Mean square error
NGTDM: Neighborhood gray tone difference matrix
OC: Ovarian cancer
O-RADS: Ovarian-Adnexal Reporting and Data System
PE: Peak enhancement
PET/CT: Positron emission tomography computed tomography
RF: Random forest
RI: Resistance index
ROC: Receiver operating characteristic curve
ROI: Region of interest
SVM: Support vector machine
TIC: Time-signal intensity curve
TL: Transfer learning
TTP: Time to peak
TVUS: Transvaginal ultrasound
US: Ultrasound
WiR: Wash-in rate
