# Supplementary figures and images for: Multimodal ultrasound-based radiomics and deep learning for differential diagnosis of O-RADS 4–5 adnexal masses
Source: Cancer Imaging. 2025 May 23;25:64. doi: 10.1186/s40644-025-00883-z (PMC12100863; doi:10.1186/s40644-025-00883-z)

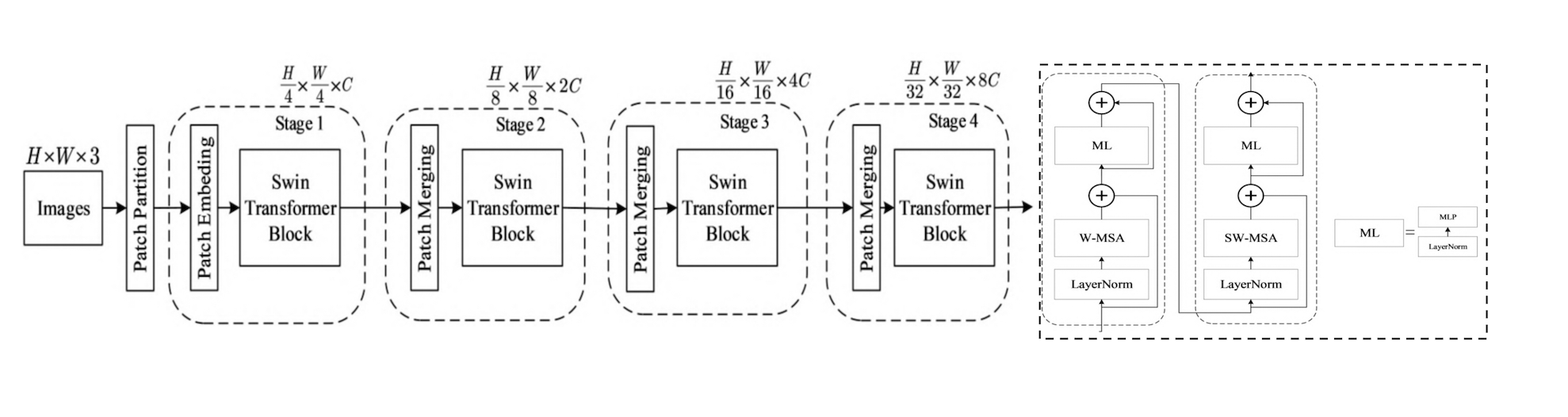

Supplement: Supplementary file 1 — Supplementary Material 1: Figure S1 The architecture of the Swin Transformer DL network. W-MSA, window multi self-attention; SW-MSA, shifted window multi self-attention; MLP, multilayer perceptron; LayerNorm, layer normalization; ML, machine learning [file 40644_2025_883_MOESM1_ESM.jpg]
