# Supplementary material for: Multimodal ultrasound-based radiomics and deep learning for differential diagnosis of O-RADS 4–5 adnexal masses
Source: Cancer Imaging. 2025 May 23;25:64. doi: 10.1186/s40644-025-00883-z (PMC12100863; doi:10.1186/s40644-025-00883-z)
Supplement: Supplementary file 5 — Supplementary Material 5: Table S1 Diagnostic performance of Clinic_models by four classifiers [file 40644_2025_883_MOESM5_ESM.docx]

| Model | Classifier | AUC | 95%CI | Accuracy | Sensitivity | Specificity | Precision | F1-score |
| --- | --- | --- | --- | --- | --- | --- | --- | --- |
| **Train** |  |  |  |  |  |  |  |  |
| Clinic | LR | 0.833 | 0.775-0.891 | 0.769 | 0.703 | 0.810 | 0.696 | 0.699 |
|  | KNN | 0.887 | 0.846-0.927 | 0.811 | 0.648 | 0.912 | 0.819 | 0.724 |
|  | GBT | 0.931 | 0.900-0.963 | 0.861 | 0.725 | 0.946 | 0.892 | 0.800 |
|  | SVM | 0.790 | 0.723-0.856 | 0.803 | 0.560 | 0.952 | 0.879 | 0.685 |
| **Test** |  |  |  |  |  |  |  |  |
| Clinic | LR | 0.848 | 0.767-0.930 | 0.775 | 0.694 | 0.818 | 0.676 | 0.685 |
|  | KNN | 0.794 | 0.699-0.888 | 0.784 | 0.583 | 0.894 | 0.750 | 0.656 |
|  | GBT | 0.809 | 0.719-0.899 | 0.794 | 0.639 | 0.879 | 0.742 | 0.687 |
|  | SVM | 0.729 | 0.607-0.850 | 0.765 | 0.528 | 0.894 | 0.731 | 0.613 |

**Table S1** Diagnostic performance of Clinic_models by four classifiers.

KNN (K-nearest neighbor), SVM (support vector machine), LR (logistic regression), RF (random forest).
