# Supplementary material for: Multimodal ultrasound-based radiomics and deep learning for differential diagnosis of O-RADS 4–5 adnexal masses
Source: Cancer Imaging. 2025 May 23;25:64. doi: 10.1186/s40644-025-00883-z (PMC12100863; doi:10.1186/s40644-025-00883-z)
Supplement: Supplementary file 6 — Supplementary Material 6: Table S2 Features selection process of Rad_2DUS model, Rad_CEUS model, and Rad_2D_CEUS model [file 40644_2025_883_MOESM6_ESM.docx]

| **Model** | **Input feature** | **Variance threshold**  **(threshold=0.75)** | **SelectKbest(p<0.05)** | **Lasso(cv=10)** | **Intercept** |
| --- | --- | --- | --- | --- | --- |
| Rad_2DUS | 846 | 674 | 426 | 21 | 0.378 |
| Rad_CEUS | 4230 | 3375 | 2436 | 17 | 0.378 |
| Rad_2DUS_CEUS | 5076 | 4049 | 2862 | 31 | 0.378 |

**Table S2** Features selection process of Rad_2DUS model, Rad_CEUS model, and Rad_2D_CEUS model.

CEUS (contrast-enhanced ultrasound), 2DUS (two-dimensional ultrasound), Rad (radiomics), LASSO (least absolute shrinkage and selection operator).
