# Supplementary material for: Multimodal ultrasound-based radiomics and deep learning for differential diagnosis of O-RADS 4–5 adnexal masses
Source: Cancer Imaging. 2025 May 23;25:64. doi: 10.1186/s40644-025-00883-z (PMC12100863; doi:10.1186/s40644-025-00883-z)
Supplement: Supplementary file 7 — Supplementary Material 7: Table S3 Diagnostic performance of Rad_2DUS model, Rad_CEUS model, and Rad_2D_CEUS model by four classifiers [file 40644_2025_883_MOESM7_ESM.docx]

**Table S3** Diagnostic performance of Rad_2DUS model, Rad_CEUS model, and Rad_2D_CEUS model by four classifiers.

| Model | Classifier | AUC | 95%CI | Accuracy | Sensitivity | Specificity | Precision | F1-score |
| --- | --- | --- | --- | --- | --- | --- | --- | --- |
| **Train** |  |  |  |  |  |  |  |  |
| Rad_2DUS | LR | 0.806 | 0.752-0.861 | 0.718 | 0.711 | 0.723 | 0.610 | 0.656 |
|  | KNN | 0.834 | 0.786-0.883 | 0.756 | 0.589 | 0.858 | 0.716 | 0.646 |
|  | GBT | 0.912 | 0.876-0.947 | 0.824 | 0.600 | 0.959 | 0.900 | 0.720 |
|  | SVM | 0.777 | 0.716-0.838 | 0.735 | 0.522 | 0.865 | 0.701 | 0.599 |
| Rad_CEUS | LR | 0.869 | 0.822-0.916 | 0.807 | 0.800 | 0.811 | 0.720 | 0.758 |
|  | KNN | 0.905 | 0.869-0.941 | 0.836 | 0.733 | 0.899 | 0.815 | 0.772 |
|  | GBT | 0.952 | 0.928-0.975 | 0.857 | 0.778 | 0.905 | 0.833 | 0.805 |
|  | SVM | 0.821 | 0.759-0.883 | 0.819 | 0.711 | 0.885 | 0.790 | 0.749 |
| Rad_2DUS_CEUS | LR | 0.893 | 0.852-0.933 | 0.794 | 0.800 | 0.791 | 0.699 | 0.746 |
|  | KNN | 0.910 | 0.876-0.945 | 0.828 | 0.744 | 0.878 | 0.788 | 0.766 |
|  | GBT | 0.944 | 0.917-0.970 | 0.870 | 0.811 | 0.905 | 0.839 | 0.825 |
|  | SVM | 0.859 | 0.807-0.911 | 0.828 | 0.722 | 0.892 | 0.802 | 0.760 |
| **Test** |  |  |  |  |  |  |  |  |
| Rad_2DUS | LR | 0.737 | 0.634-0.839 | 0.686 | 0.676 | 0.692 | 0.556 | 0.610 |
|  | KNN | 0.708 | 0.603-0.813 | 0.696 | 0.541 | 0.785 | 0.588 | 0.563 |
|  | GBT | 0.706 | 0.603-0.808 | 0.657 | 0.378 | 0.815 | 0.538 | 0.444 |
|  | SVM | 0.724 | 0.614-0.835 | 0.716 | 0.486 | 0.846 | 0.643 | 0.554 |
| Rad_CEUS | LR | 0.826 | 0.737-0.916 | 0.765 | 0.757 | 0.769 | 0.651 | 0.700 |
|  | KNN | 0.815 | 0.729-0.902 | 0.735 | 0.595 | 0.815 | 0.647 | 0.620 |
|  | GBT | 0.795 | 0.704-0.885 | 0.765 | 0.676 | 0.815 | 0.676 | 0.676 |
|  | SVM | 0.826 | 0.735-0.917 | 0.794 | 0.676 | 0.862 | 0.735 | 0.704 |
| Rad_2DUS_CEUS | LR | 0.842 | 0.758-0.926 | 0.794 | 0.757 | 0.815 | 0.700 | 0.727 |
|  | KNN | 0.831 | 0.742-0.920 | 0.784 | 0.649 | 0.862 | 0.727 | 0.686 |
|  | GBT | 0.819 | 0.730-0.909 | 0.775 | 0.649 | 0.846 | 0.706 | 0.676 |
|  | SVM | 0.822 | 0.729-0.914 | 0.814 | 0.649 | 0.908 | 0.800 | 0.716 |

CEUS (contrast-enhanced ultrasound), 2DUS (two-dimensional ultrasound), Rad (radiomics), KNN (K-nearest neighbor), SVM (support vector machine), LR (logistic regression), RF (random forest), AUC (area under the receiver operating characteristic curve).
