# Supplementary material for: Multimodal ultrasound-based radiomics and deep learning for differential diagnosis of O-RADS 4–5 adnexal masses
Source: Cancer Imaging. 2025 May 23;25:64. doi: 10.1186/s40644-025-00883-z (PMC12100863; doi:10.1186/s40644-025-00883-z)
Supplement: Supplementary file 8 — Supplementary Material 8: Table S4 Diagnostic performance of DL_2DUS model, DL_CEUS model, and DL_2D_CEUS model [file 40644_2025_883_MOESM8_ESM.docx]

**Table S4** Diagnostic performance of DL_2DUS model, DL_CEUS model, and DL_2D_CEUS model.

| Model | AUC | 95%CI | Accuracy | Sensitivity | Specificity | Precision | F1-score |
| --- | --- | --- | --- | --- | --- | --- | --- |
| **Train** |  |  |  |  |  |  |  |
| DL_2DUS | 0.984 | 0.967-1.000 | 0.954 | 0.922 | 0.973 | 0.954 | 0.938 |
| DL_CEUS | 0.997 | 0.990-1.000 | 0.983 | 1.000 | 0.973 | 0.957 | 0.978 |
| DL_2DUS_CEUS | 1.000 | 1.000-1.000 | 1.000 | 1.000 | 1.000 | 1.000 | 1.000 |
| **Test** |  |  |  |  |  |  |  |
| DL_2DUS | 0.793 | 0.697-0.889 | 0.745 | 0.622 | 0.815 | 0.657 | 0.639 |
| DL_CEUS | 0.823 | 0.744-0.902 | 0.657 | 0.973 | 0.477 | 0.514 | 0.673 |
| DL_2DUS_CEUS | 0.828 | 0.750-0.906 | 0.706 | 0.892 | 0.600 | 0.559 | 0.687 |

CEUS (contrast-enhanced ultrasound), 2DUS (two-dimensional ultrasound), DL (deep learning), AUC (area under the receiver operating characteristic curve).
