# Supplementary material for: Multimodal ultrasound-based radiomics and deep learning for differential diagnosis of O-RADS 4–5 adnexal masses
Source: Cancer Imaging. 2025 May 23;25:64. doi: 10.1186/s40644-025-00883-z (PMC12100863; doi:10.1186/s40644-025-00883-z)
Supplement: Supplementary file 9 — Supplementary Material 9: Table S5 Features selection process of Rad_DL_2DUS_CEUS model [file 40644_2025_883_MOESM9_ESM.docx]

| **Model** | **Input feature** | **Variance threshold**  **(threshold=0.75)** | **SelectKbest (p<0.05)** | **Lasso**  **(cv=10)** | **Intercept** |
| --- | --- | --- | --- | --- | --- |
| Rad_DL_2DUS_CEUS | 9684 | 8657 | 4425 | 77 | 0.378 |

**Table S5** Features selection process of Rad_DL_2DUS_CEUS model.

CEUS (contrast-enhanced ultrasound), 2DUS (two-dimensional ultrasound), Rad (radiomics), DL (deep learning).
