# Supplementary material for: Multimodal ultrasound-based radiomics and deep learning for differential diagnosis of O-RADS 4–5 adnexal masses
Source: Cancer Imaging. 2025 May 23;25:64. doi: 10.1186/s40644-025-00883-z (PMC12100863; doi:10.1186/s40644-025-00883-z)
Supplement: Supplementary file 10 — Supplementary Material 10: Table S6 Diagnostic performance of Rad_DL_2D_CEUS models by four classifiers [file 40644_2025_883_MOESM10_ESM.docx]

| Model | Classifier | AUC | 95%CI | Accuracy | Sensitivity | Specificity | Precision | F1-score |
| --- | --- | --- | --- | --- | --- | --- | --- | --- |
| **Train** |  |  |  |  |  |  |  |  |
| Rad_DL_2DUS_CEUS | LR | 0.993 | 0.984-1.000 | 0.966 | 0.956 | 0.973 | 0.956 | 0.956 |
|  | KNN | 0.997 | 0.993-1.000 | 0.966 | 0.956 | 0.973 | 0.956 | 0.956 |
|  | GBT | 1.000 | 1.000-1.000 | 1.000 | 1.000 | 1.000 | 1.000 | 1.000 |
|  | SVM | 0.994 | 0.986-1.000 | 0.966 | 0.956 | 0.973 | 0.956 | 0.956 |
| **Test** |  |  |  |  |  |  |  |  |
| Rad_DL_2DUS_CEUS | LR | 0.927 | 0.875-0.979 | 0.863 | 0.892 | 0.846 | 0.767 | 0.825 |
|  | KNN | 0.894 | 0.828-0.961 | 0.853 | 0.838 | 0.862 | 0.775 | 0.805 |
|  | GBT | 0.909 | 0.848-0.969 | 0.853 | 0.838 | 0.862 | 0.775 | 0.805 |
|  | SVM | 0.907 | 0.842-0.972 | 0.853 | 0.865 | 0.846 | 0.762 | 0.810 |

**Table S6** Diagnostic performance of Rad_DL_2D_CEUS models by four classifiers.

CEUS (contrast-enhanced ultrasound), 2DUS (two-dimensional ultrasound), Rad (radiomics), DL (deep learning), KNN (K-nearest neighbor), SVM (support vector machine), LR (logistic regression), RF (random forest), AUC (area under the receiver operating characteristic curve).
