# Supplementary material for: Multimodal ultrasound-based radiomics and deep learning for differential diagnosis of O-RADS 4–5 adnexal masses
Source: Cancer Imaging. 2025 May 23;25:64. doi: 10.1186/s40644-025-00883-z (PMC12100863; doi:10.1186/s40644-025-00883-z)
Supplement: Supplementary file 11 — Supplementary Material 11: Table S7 Diagnostic performance of Clinic_Rad_DL models by four classifiers [file 40644_2025_883_MOESM11_ESM.docx]

| Model | Classifier | AUC | 95%CI | Accuracy | Sensitivity | Specificity | Precision | F1-score |
| --- | --- | --- | --- | --- | --- | --- | --- | --- |
| **Train** |  |  |  |  |  |  |  |  |
| Clinic_Rad_DL | LR | 0.991 | 0.982-1.000 | 0.962 | 0.956 | 0.966 | 0.946 | 0.951 |
|  | KNN | 0.995 | 0.990-1.000 | 0.966 | 0.934 | 0.986 | 0.977 | 0.955 |
|  | GBT | 1.000 | 1.000-1.000 | 1.000 | 1.000 | 1.000 | 1.000 | 1.000 |
|  | SVM | 0.994 | 0.986-1.000 | 0.971 | 0.956 | 0.980 | 0.967 | 0.961 |
| **Test** |  |  |  |  |  |  |  |  |
| Clinic_Rad_DL | LR | 0.929 | 0.877-0.980 | 0.853 | 0.889 | 0.833 | 0.744 | 0.810 |
|  | KNN | 0.896 | 0.831-0.960 | 0.853 | 0.861 | 0.848 | 0.756 | 0.805 |
|  | GBT | 0.909 | 0.852-0.966 | 0.853 | 0.861 | 0.848 | 0.756 | 0.805 |
|  | SVM | 0.874 | 0.797-0.951 | 0.843 | 0.861 | 0.833 | 0.738 | 0.795 |

**Table S7** Diagnostic performance of Clinic_Rad_DL models by four classifiers.

Rad (radiomics), DL (deep learning), KNN (K-nearest neighbor), SVM (support vector machine), LR (logistic regression), RF (random forest), AUC (area under the receiver operating characteristic curve).
