# Supplementary material for: Multimodal ultrasound-based radiomics and deep learning for differential diagnosis of O-RADS 4–5 adnexal masses
Source: Cancer Imaging. 2025 May 23;25:64. doi: 10.1186/s40644-025-00883-z (PMC12100863; doi:10.1186/s40644-025-00883-z)
Supplement: Supplementary file 12 — Supplementary Material 12: Table S8 Features and coefficients of Clinic_model and Clinic_Rad_DL model [file 40644_2025_883_MOESM12_ESM.docx]

**Table S8** Features and coefficients of Clinic_model and Clinic_Rad_DL model.

| Clinic_model | | Clinic_Rad_DL model | |
| --- | --- | --- | --- |
| Features | coefficients | Features | coefficients |
| CA125 | 0.099328567 | square_firstorder_90Percentile | 0.051771613 |
| edge | 0.096478168 | wavelet-L_gldm_SmallDependenceLowGrayLevelEmphasis | -0.014936339 |
| HE4 | 0.067902492 | square_firstorder_Skewness | -0.027618247 |
| CA724 | 0.047687548 | exponential_firstorder_Skewness | -0.04664539 |
| MeanLin2 | 0.014115132 | wavelet-H_firstorder_Energy | 0.038717815 |
| PE1 | 0.056498979 | wavelet-L_glcm_ClusterShade | -0.01676532 |
| PE2 | 0.02079272 | lbp-2D_firstorder_10Percentile | -0.030228629 |
| size | 0.035269777 | original_glcm_ClusterProminence | -0.029705273 |
| CEA | 0.060650271 | wavelet-L_ngtdm_Contrast | -0.004712708 |
| TTP1 | -0.035967686 | original_glcm_MCC | -0.020805221 |
| age | 0.013411671 | logarithm_glcm_MCC | -7.46E-18 |
| CA125 | 0.099328567 | squareroot_glcm_MCC | -7.46E-18 |
| edge | 0.096478168 | exponential_glrlm_ShortRunEmphasis | -0.054064957 |
| HE4 | 0.067902492 | exponential_glrlm_GrayLevelNonUniformity | 0.008029926 |
| CA724 | 0.047687548 | lbp-2D_glszm_GrayLevelNonUniformity | 7.46E-18 |
|  |  | square_glrlm_GrayLevelNonUniformity | 7.46E-18 |
|  |  | square_glszm_GrayLevelNonUniformity | 3.73E-18 |
|  |  | original_shape2D_PerimeterSurfaceRatio | -0.066181929 |
|  |  | wavelet-H_firstorder_Median | 0.053621757 |
|  |  | original_glcm_Imc1 | 0.013606413 |
|  |  | logarithm_glcm_Imc1 | 7.46E-18 |
