# Supplementary material for: Multimodal ultrasound-based radiomics and deep learning for differential diagnosis of O-RADS 4–5 adnexal masses
Source: Cancer Imaging. 2025 May 23;25:64. doi: 10.1186/s40644-025-00883-z (PMC12100863; doi:10.1186/s40644-025-00883-z)
Supplement: Supplementary file 13 — Supplementary Material 13: Table S9 Diagnostic performance of Clinic_model, Rad_2D_CEUS model, DL_2D_CEUS model, Rad_DL_2D_CEUS model, and Clinic_Rad_DL model [file 40644_2025_883_MOESM13_ESM.docx]

| Model | AUC | 95%CI | Accuracy | Sensitivity | Specificity | Precision | F1-score |
| --- | --- | --- | --- | --- | --- | --- | --- |
| **Train** |  |  |  |  |  |  |  |
| Clinic | 0.833 | 0.775-0.891 | 0.769 | 0.703 | 0.810 | 0.696 | 0.699 |
| Rad_2DUS_CEUS | 0.893 | 0.852-0.933 | 0.794 | 0.800 | 0.791 | 0.699 | 0.746 |
| DL_2DUS_CEUS | 1.000 | 1.000-1.000 | 1.000 | 1.000 | 1.000 | 1.000 | 1.000 |
| Rad_DL_2DUS_CEUS | 0.993 | 0.984-1.000 | 0.966 | 0.956 | 0.973 | 0.956 | 0.956 |
| Clinic_Rad_DL | 0.991 | 0.982-1.000 | 0.962 | 0.956 | 0.966 | 0.946 | 0.951 |
| **Test** |  |  |  |  |  |  |  |
| Clinic | 0.848 | 0.767-0.930 | 0.775 | 0.694 | 0.818 | 0.676 | 0.685 |
| Rad_2DUS_CEUS | 0.842 | 0.758-0.926 | 0.794 | 0.757 | 0.815 | 0.700 | 0.727 |
| DL_2DUS_CEUS | 0.828 | 0.750-0.906 | 0.706 | 0.892 | 0.600 | 0.559 | 0.687 |
| Rad_DL_2DUS_CEUS | 0.927 | 0.875-0.979 | 0.863 | 0.892 | 0.846 | 0.767 | 0.825 |
| Clinic_Rad_DL | 0.929 | 0.877-0.980 | 0.853 | 0.889 | 0.833 | 0.744 | 0.810 |

**Table S9** Diagnostic performance of Clinic_model, Rad_2D_CEUS model, DL_2D_CEUS model, Rad_DL_2D_CEUS model, and Clinic_Rad_DL model.

CEUS (contrast-enhanced ultrasound), 2DUS (two-dimensional ultrasound), Rad (radiomics), DL (deep learning), AUC (area under the receiver operating characteristic curve).
