# Supplementary material for: Multimodal ultrasound-based radiomics and deep learning for differential diagnosis of O-RADS 4–5 adnexal masses
Source: Cancer Imaging. 2025 May 23;25:64. doi: 10.1186/s40644-025-00883-z (PMC12100863; doi:10.1186/s40644-025-00883-z)
Supplement: Supplementary file 14 — Supplementary Material 14: Table S10 The DeLong test of models [file 40644_2025_883_MOESM14_ESM.docx]

| Model | Clinic | Rad_2DUS_CEUS | DL_2DUS_CEUS | Rad_DL_2DUS_CEUS | Clinic_Rad_DL |
| --- | --- | --- | --- | --- | --- |
| **Train** |  |  |  |  |  |
| Clinic | 1 | 0.044 | <0.05 | <0.05 | <0.05 |
| Rad_2DUS_CEUS |  | 1 | <0.05 | <0.05 | <0.05 |
| DL_2DUS_CEUS |  |  | 1 | <0.05 | <0.05 |
| Rad_DL_2DUS_CEUS |  |  |  | 1 | 0.337 |
| Clinic_Rad_DL |  |  |  |  | 1 |
| **Test** |  |  |  |  |  |
| Clinic | 1 | 0.599 | <0.05 | 0.164 | <0.05 |
| Rad_2DUS_CEUS |  | 1 | <0.05 | <0.05 | <0.05 |
| DL_2DUS_CEUS |  |  | 1 | <0.05 | <0.05 |
| Rad_DL_2DUS_CEUS |  |  |  | 1 | 0.394 |
| Clinic_Rad_DL |  |  |  |  | 1 |

**Table S10** The DeLong test of models.

CEUS (contrast-enhanced ultrasound), 2DUS (two-dimensional US), Rad (radiomics), DL (deep learning).
